# Quantitative Estimation of the Nonstationary Behavior of Neural Spontaneous Activity

**DOI:** 10.1155/2010/785919

**Published:** 2009-12-10

**Authors:** João-Batista Destro-Filho, Carlos-Alberto Estombelo-Montesco, Luiz-Otavio Murta-Junior, Sergio Martinoia, Michela Chiappalone, Suelen Moreira-Marques, Amanda F. Neves

**Affiliations:** ^1^Biomedical Engineering Laboratory (BioLab), School of Electronic Engineering (FEELT), Federal University of Uberlândia (UFU), Av. João Naves de Avila 2121, Santa Mônica, 38400-902 Uberlândia, MG, Brazil; ^2^Physics Department, FFCLH, São Paulo University (USP), Av. Bandeirantes 3900.14040-901 Ribeirão Preto, Brazil; ^3^Neuroengineering & Bio-Nano Technology Group, Department of Biophysical and Electronic Engineering, University of Genova, Via Opera Pia 11A, 16145 Genova, Italy

## Abstract

The “stationarity time” (ST) of neuronal spontaneous activity signals of rat embryonic cortical cells, measured by means of a planar Multielectrode Array (MEA), was estimated based on the “Detrended Fluctuation Analysis” (DFA). The ST is defined as the mean time interval during which the signal under analysis keeps its statistical characteristics constant. An upgrade on the DFA method is proposed, leading to a more accurate procedure. Strong statistical correlation between the ST, estimated from the Absolute Amplitude of Neural Spontaneous Activity (AANSA) signals and the Mean Interburst Interval (MIB), calculated by classical spike sorting methods applied to the interspike interval time series, was obtained. In consequence, the MIB may be estimated by means of the ST, which further includes relevant biological information arising from basal activity. The results point out that the average ST of MEA signals lies between 2-3 seconds. Furthermore, it was shown that a neural culture presents signals that lead to different statistical behaviors, depending on the relative geometric position of each electrode and the cells. Such behaviors may disclose physiological phenomena, which are possibly associated with different adaptation/facilitation mechanisms.

## 1. Introduction

The digital processing of biological signals may be considered a challenging task [1], due to the underlying characteristics of such systems and signals: the nonlinearity, which is closely connected with the complex behavior of the alive organisms [2, 3]; and the nonstationarity of the time series [4].

A classical mathematical procedure in neuronal signal processing consists of the detection of spikes connected with action potentials, which requires the establishment of an amplitude threshold, above which any potential is considered a spike [5]. The next step is devoted to the estimation of the “Interspike Interval” (ISI) time series, including spike classification [6], which enables several analyses in the field of neuronal coding [3]. Notice that spike classification is based on pattern recognition theory, involving tools such as Mahalanobis minimum distance [6, 7] and Independent Component Analysis [5]. In addition, neural connectivity [8] is also a very important research field, based on the application of cross-correlation theory [9–12] and spectral coherence [13] to the ISI time series, in order to evaluate the network of synaptic connections among cells within the cultured tissue. All these signal processing techniques are based on the concept of “ISI time series” [8], the estimation of which depends on the performance of spike detection and classification. However, to our knowledge, literature associated with all the research topics previously discussed devotes few efforts on two relevant computational issues, which establish bounds on the performance of current neurophysiological data acquisition systems: ISI time series windowing and real-time processing [14], pointing out that both of them should consider the nonstationary behavior of biological signals [4, 14].

Multielectrode Arrays (MEAs) emerged during the 1990's, in order to measure the electrical activity of cultured neurons [15]. This new approach was important to support the development of deeper studies of ISI time series, leading to significant contributions to neuronal coding theory, as well as on the effects of induced neurostimulation in neural cultures [6].

On the other hand, neuropathologies may be considered relevant deseases from a clinical viewpoint. Particularly, epilepsy disturbs 1% of the world population, corresponding to 50 million people. From this amount, at least the seizures of 30% of patients can not be well managed by conventional treatments based on anticonvulsivant drugs [2]. Henceforth, the development of new treatments is necessary, such as neuroprostheses [15, 16]. Studies using MEAs are very promising because they can provide a basis for the implementation of these technologies in a near future. 

Consequently, MEA devices should be capable to process both cellular-level signals, such as action potentials, as well as electroencephalographic (EEG) data in real time, to minimize epileptic seizures [15, 17], working as neuroprostheses. The last application surely imposes restrictions on signal processing tools. In fact, algorithms must present low computational complexity, in order to allow the lowest power dissipation [15], assuring the biocompatibility of the device [18]. Furthermore, the clinical efficiency of the neuroprosthesis-based therapy involves real-time operation [16], which must be achieved by the device. 

For these reasons, the neuroprosthesis implementation requires simple statistical tools of low computational complexity, leading to real-time signal processing. To our knowledge, these practical constraints have been very fewly addressed by literature connected with MEA-signal analysis, especially regarding the estimation of optimal data windowing, taking into consideration the non-stationary behavior of the signal. Notice that such procedure is essential for any operation linked to the MEA-signal processing [9]. Moreover, it should be important to develop mathematical tools capable of analyzing the signal to avoid the spike detection. In consequence, spike pre-processing would not be mandatory, leading to a more simple system, which of course agrees with the idea of real-time operation and low power dissipation.

A possible strategy to establish optimal windowing is based on the concept of “Stationarity Time” (ST), defined as the time interval during which the signal measured by MEA keeps its statistical characteristics constant [19]. In this context, the “Detrended Fluctuation Analysis” (DFA) may be pointed out, since it is a classical tool for the study of non-stationarity, firstly used in order to carry out the similarity analysis among animal nucleotides [20]. Later, it was employed to study the stationary behavior of neural signals in [19], in which the ST is estimated based on the visual analysis of the plot involving the average variance (*F*) as a function of the window length (*L*). In this case, “variance” regards the error calculated between the signal under analysis and its polynomial approximation, based on the principles of fractal theory. In addition, the plot *F* × *L* is fitted to a particular “alfa-exponential” spectrum [19]. Both operations of visual analysis and spectrum fitting may be considered empirical, as well as dependent on the researcher and on the application. 

In brief, the study of the stationary behavior of MEA signals can disclose important features of culture neurodynamics, as well as enabling the definition of optimal windowing, which is mandatory for efficient neuroprosthesis implementation. In this context, this paper develops the estimation of the ST of a set of spontaneous activity signals. An upgrade on DFA technique is proposed, leading to an accurate tool that is able to process the absolute amplitudes of neuronal spontaneous activity. The results are compared to classical quantities that are currently used to characterize the culture dynamics, such as the Mean-Interburst Interval, pointing out strong statiscal correlations. Finally, the neurodynamics of the culture is discussed in terms of the ST diagram, leading to physiological interpretations of the results.

## 2. Materials and Methods

### 2.1. Data Acquisition

MEA signals are characterized by the absolute amplitude of neural spontaneous activity (AANSA), collected from primary cultures of cortical neurons, extracted from rat embryos of 17-18 days. These cells were plated on planar MEAs containing 60 microelectrodes (*MEA60, Multichannel Systems, Reutlingen, Germany*). Electrode diameter is 30 *μ*m, and interelectrode spacing is 200 *μ*m. Experiments were performed after 18–34 days in vitro (DIV), as the cultures may be considered stable, in order to allow the maturation of the synapses among the neurons, supposing 4-milliseconds sampling interval. Four consecutive experiments were accomplished, each one lasting 5 minutes, leading to a continuous global sample of 20 minutes. Additional details are presented in [21].

### 2.2. Spike Analysis

MEA signals were analyzed by means of the plataform *SpikeManager* [21], which performs classical spike analysis. ISI time series were calculated, and average statistics were performed over all the sixty channels considering all the four experiments, supposing the same parameter set for spike analysis as reported in [21].

### 2.3. Classical DFA

The following quantities are defined through (1) and (2), wherein the input signal *I*, (*k*) is the absolute amplitude of the MEA signal, measured in V:

(1) $$\begin{array}{c} y\left(n\right)=\overset{N}{\underset{k=1}{\sum }}\left[I\left(k\right)-I_{\text{ave}}\right], \end{array}$$

(2) $$\begin{array}{c} I_{\text{ave}}=\;\;\frac{1}{N}\;.\;\overset{N}{\underset{k=1}{\sum }}I\left(k\right), \end{array}$$

where (*k*), (*n*) represent the discrete time; (*N*) is the length of the time series; Iave the average value of the amplitudes of MEA signal under analysis.

DFA method [19] requires three steps. Based on signal *I*(*k*), the first step calculates the parameter Iave, defined in (2), in order to generate *y*(*n*), as presented in (1), which can be considered the zero-mean MEA signal. 

In the second step, the signal *y*(*n*) is divided up into consecutive and nonoverlapping segments of *L* length, so that 100 < *L* < 15000. Afterwards, a polynomial fit *T i*(*n*) is carried out in order to approach the signal *y*(*n*), where index *i* represents the segment under analysis. This fit is called “local trend”, which is defined in the following equations:

(3) $$\begin{array}{cc} Ti\left(n\right) & =A^{*}n+B,\\ Ci\left(n,L\right) & =y\left(n\right)-Ti\left(n\right), \end{array}$$

where *A*, *B* are real constants, estimated as a function of *y(n)* values within segment *i*; *Ci(n*, *L) *is the “detrended walk”, associated with *y(n)*, which depends on the length *L* of each segment. 

It should be noticed that (3) present a linear regression, which will be considered in this paper, since this approach leads to a reasonable trade-off between algorithm performance and low computational complexity, according to comparative studies accomplished in [19].

In the third step, the detrended walk variance is calculated for each segment, and, finally, all these variances are averaged, considering all segments, as shown by (4) as follows:

(4) $$F\left(L\right)=\sqrt{\frac{1}{N+L-1}\;\;\overset{N-L+1}{\underset{i=1}{\sum }}\overset{L}{\underset{k=1}{\sum }}{\left(C_{i}\left(n,L\right)\right)}^{2}},$$

where the parameter *F*(*L*) represents the average variance of the residual error among the signal *y*(*n*) and the local trend *T i*(*n*), as a function of the window length *L*.

**Figure 1:**
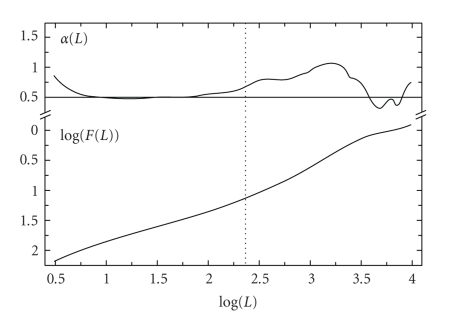
Example of the classical methodology for ST estimation through DFA [19]. The lower plot shows log (*F*(*L*)) × log (*L*); *α*(*L*) is presented in the upper plot. The vertical dashed line in the middle of the figure represents the signal rupture detected by the technique in [19].

In the following, the classical methodology for the ST estimation based on the variance *F*(*L*) is discussed. Figure 1 presents an example of the plot log  10(*F*(*L*)) × log  10(*L*), which is depicted in the lower part of the graph; whereas, the first derivative of *F*(*L*) with respect to *L* is depicted in the upper part, by the plot *α*(*L*) × log  10(*L*). Notice that the first derivative is also called “angular variation *α*(*L*)”. It should be pointed out that as *L* increases the slope of the function, *F*(*L*) keeps constant up to a specific point, wherein the slope *α*(*L*) increases or decreases considerably, leading to peaks in the *α*(*L*) amplitude. This sudden change in the derivative amplitude is called “signal rupture” [19], which is closely connected with the signal nonstationarity as discussed in [4].

The ST is estimated as the *L* value of plot log 10(F(*L*)) × log  10(*L*) associated with the signal rupture [4, 9], which may be established based on a careful analysis of *α*(*L*) variations. Generally, the signal rupture is considered at a specific value of *L* wherein the first derivative is not constant. This value is located between two regions of constant amplitude of *α (L) *graph. In the case under analysis, Figure 1 points out two regions of constant first derivative: the first one lies within log(*L*) <2.0 followed by the second region log(*L*) >2.5. Henceforth, the ST is estimated as a value within the interval 2.0 < log(*L*) <2.5, and the signal rupture is established by the vertical dashed line in Figure 1.

The ST characterizes the signal time-variation profile as discussed in the following. Since ST is estimated as particular value of the window length *L*, (4) points out that high-amplitude values of ST are associated with the detection of a small number of ruptures, leading to few statistical-behavior variations, which in turn characterizes a stationary signal. Conversely, the smaller the values of ST, the larger will be the amount of ruptures, which characterizes a non-stationary behavior.

In brief, the ST estimation procedure previously described [4, 19] does not follow a rigorous mathematical methodology, so that results depend on the signal under analysis and on the researcher goals. In addition, notice from Figure 1 that other ruptures should take place at other window lengths different from log  10(*L*) = 2.4 seconds. Although these ruptures do point out statistical changes, they are not taken into consideration in the classical method. For all these reasons, a more accurate mathematical technique for ST estimation should be developed, including all the non-stationary points of the signal, which are presented in the following.

### 2.4. A New Approach for ST Estimation Based on DFA

In order to highlight all the changes of the first derivative of *F(L)* plot , the concept of the second derivative of function *F(L*) with respect to variable *L* was employed, according to the following equations:

(5) $$D\left(i+1\right)=\frac{F\left(L\right)\left(i+1\right)-F\left(L\right)\left(i\right)}{L\left(i+1\right)-L\left(i\right)},$$

where *i* is the parameter that represents the *i*th element of vector *F(L)*; *D* is the first derivative of *F*(*L*); *F*(*L*)(*i* + 1)−*F*(*L*)(*i*) is the variation of *F*(*L*); *L*(*i* + 1)−*L*(*i*) is the variation of the window length.

(6) $$D2\left(i+1\right)=\frac{D\left(i+1\right)-D\left(i\right)}{L\left(i+1\right)-L\left(i\right)},$$

where *D*2 is the second derivative; *D*(*i* + 1) − *D*(*i*) is the variation of the first derivative of *F(L).*

**Figure 2:**
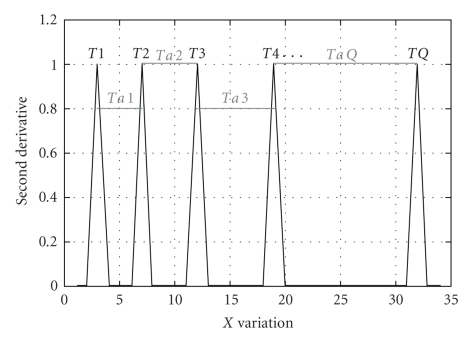
Hypothetical illustration of a second-derivative generic plot, associated with a function log  10(*F*(*L*)) × log  10(*L*). Vertical axis depicts the amplitude of *D*2, as defined in (6); whereas, horizontal axis presents the values of *L*. Signal rupture points take place exactly at times {*T*(1),…, *T*(*Q*)}, for which function *D*2 attains a peak of amplitude 1. The stationarity intervals {*Ta*(1),…, *Ta*(*Q* − 1)} are used to estimate the final *ST* of a single MEA channel, based on (7).

The ST estimation for one single electrode is performed based on the plot *D*2 × *L*, which will be discussed in the following by means of Figure 2. The peaks of this figure indicate strong variations of *D*, the first derivative of *F*(*L*), thus pointing out that ruptures are taking place for window lengths *L* = {*T*(1), *T*(2), *T*(3)…*T*(*Q*)}, wherein *Q* is the number of ruptures. The symbol *T* is used to indicate a time associated with the window *L*, since *T* is equal to *L* multiplied by the sampling rate. Notice that, based on the properties of the second derivative, just positive peaks in function *D*2 are of interest. In consequence, the signal may be considered stationary during the time intervals *Ta*(1) = *T*(2) − *T*(1); *Ta*(2) = *T*(3) − *T*(2); *Ta*(3) = *T*(4) − *T*(3);…; *Ta*(*Q* − 1) = *T*(*Q*) – *T*(*Q* − 1). Henceforth, there is a set of stationarity times {*Ta*(1), *Ta*(2), *Ta*(3),…, *Ta*(*Q* − 1)}, so that the final ST is estimated as the weighted average of all times in the set, as defined in the following equation:

(7) $$ST=\frac{q{\left(1\right)}^{*}Ta\left(1\right)\;\;+\cdots \;+\;\;q{\left(Q-1\right)}^{*}Ta\left(Q-1\right)}{q\left(1\right)+q\left(2\right)+\cdots +\;\;q\left(Q-1\right)},$$

where {*q*(1),…, *q*(*Q*)} is the number of occurences of a specific time interval *Ta(i)*; *i* = 1,2,3,…, *Q*− 1 respectively. 

Considering just one single five-minute experiment, the average ST of each electrode was estimated based on (7). The representative ST value associated with the ensemble of 64 electrodes, for this same single experiment, was calculated based on the average considering all the mean STs, each of them characterizing one single electrode. Finally, an overall medium ST was estimated through the average performed on the four representative ST values, each of them connected to a single experiment.

## 3. Results

Table 1 presents a general characterization of the culture under study, following classical spike analysis.

**Figure 3:**
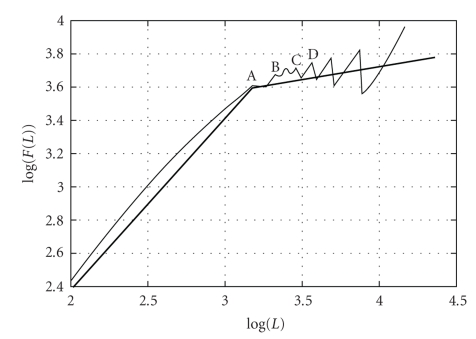
Overall average log(*F*(*L*)) function resulting from the application of the proposed DFA method is depicted as the thin line, considering all 64 electrodes and the four experiments. The horizontal axis is measured in seconds. The thick line represents an approximation generally performed by literature [9, 19]; ST = 3.2 seconds.

The processing of all MEA channels, considering all the four experiments, leads to the estimation of the overall log(*F*(*L*)) function based on (4), which is shown by the thin-line plot in Figure 3. Points A, B, C, and D of this plot clearly indicate that strong ruptures have taken place. The thick line in Figure 3 represents a rough and simple linear approach of the real log (*F*(*L*)) × *L* plot based on two straight lines, that is, generally performed in order to estimate the ST as the value of *L* for which the two straight lines meet each other [19, 20]. In consequence, based on this approach, the ST of Figure 3 is estimated as *L* = 3.2 seconds. Of course, such approach does not consider points A, B, C, D, which may highlight important characteristics of the signal.

**Figure 4:**
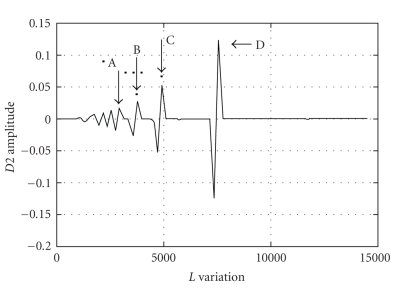
Overall average value of *D*2(*i*) × *L*, based on the results of Figure 3, wherein the horizontal axis presents the window length *L* measured as the number of samples, and the vertical axis is measured in absolute values. The points of the plot identified by letters A, B, C, D correspond to the same points A, B, C, D of Figure 3.

The second derivative *D2(i)* of the function depicted in Figure 3 was estimated through the application of (5) and (6), leading to the plot *D*2 × *L* presented in Figure 4. Of course, this last figure clearly points out the values of *L* associated to signal ruptures, which are completely disregarded by the classical approach [9, 19] based on the linear approximation (see thick line in Figure 3). Based on Figures 3 and 4, the ST estimation should consider strong signal statistical variations at 3.2 seconds (point A), 3.3 seconds (point B), 3.4 seconds (point C), and 3.6 seconds (point D). If one considers the ST the average among all these values [19], then ST_2_ = 3.35 seconds, which is slightly different from ST = 3.2 seconds estimated by the classical approach (see Figure 3).

Table 2 summarizes the results obtained for each five-minute experiment, presenting values that arise from averaging all the sixty microelectrodes. Results involve both ST estimated based on (7), which was applied to AANSA signals and to ISI time series, as well as characteristic times associated to the general interspike and interburst interval histograms, which were estimated by classical spike processing methods on the *SpikeManager* [21] platform (see subsection “Spike Analysis” in section “Materials and Methods”).

**Figure 5:**
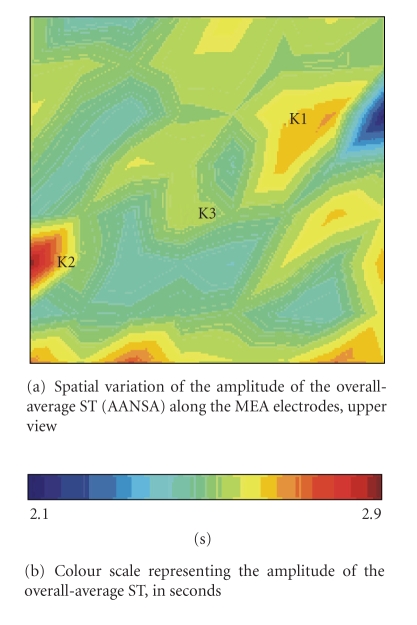


Figure 5 presents a bidimensional visualization of the overall-ST amplitude variation along the MEA device. The horizontal and vertical axes of Figure 5(a) present the spatial coordinates associated with the position of each electrode, and the color scale of Figure 5(b) provide the overall ST amplitudes, measured in seconds, thus considering the average performed on all the results obtained on the four experiments.

**Figure 6:**
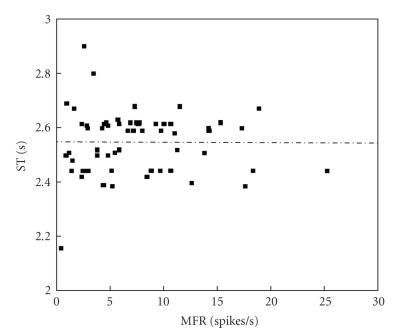
Dispersion plot involving ST and MFR for 60 channels, four experiments; *r* = −0.0111, *P* = 0.9334.

**Figure 7:**
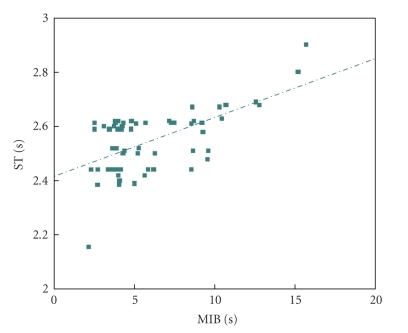
Dispersion plot involving ST and MIB for 60 channels, four experiments; *r* = 0.5956, *P* < .0001.

**Figure 8:**
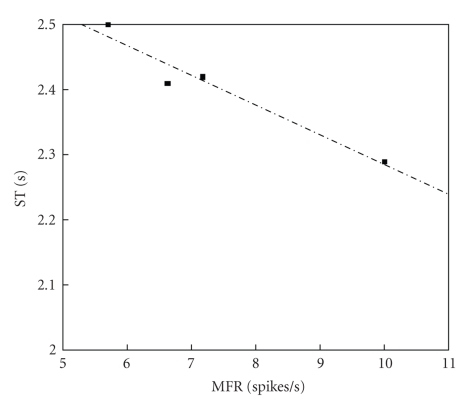
Dispersion plot involving ST and MFR, average for 60 channels, four experiments; *r* = −0.9721, *P* = .0279.

**Figure 9:**
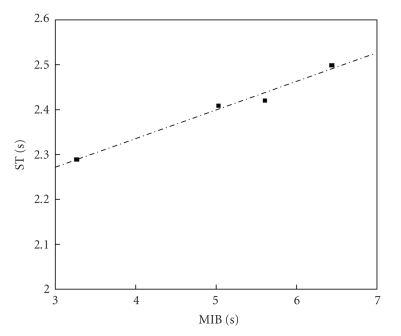
Dispersion plot involving ST and MIB, average for 60 channels, four experiments; *r* = 0.9891, *P* = .0109.

**Figure 10:**
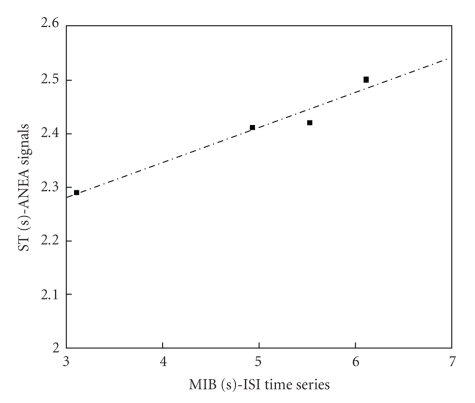
Dispersion plot involving ST estimated for AANSA signals and ST estimated for ISI time series, average for 60 channels, four experiments; *r* = 0.9792, *P* = .0208.

In order to get further insights on the relationship of the several quantities of Table 2, Pearson correlation coefficients *r* among all of them were estimated, and *t*-student hypothesis-tests have been carried out to assess the significance of all these coefficients, supposing *α* = 0.05. Figures 6 and 7 present results of the overall-average values for sixty channels; whereas, Figures 8, 9, and 10 depict results considering the overall-average values in terms of each experiment. Table 3 summarizes the results of these figures, also including other ones. 

## 4. Discussion

The last line of Table 2 depicts the overall-average ST = 2.40 seconds, which is different from the ST obtained by the classical approach (3.3 seconds, as discussed in Figure 3 above). Such difference points out that the proposed method is much more accurate than the rough estimation technique of Figure 3 [19, 20].

Consider now Table 2. In principle, ST estimated from AANSA signals is quite different from that estimated from ISI time series, which is not a surprising result, since the first one includes basal activity information, and the second one is just spiking information. The overall-average value of the ST estimated from AANSA signals also seems not to be related with any other spiking-analysis values of the table. However, the overall-average ST estimated for ISI time series is quite close to the overall-average MIB.

Results of Figures 6–10 and Table 3 aim at clarifying discussions of the previous paragraph based on a rigorous statistical approach. Beforehand, however, it should be recalled how to analyze these results, based on the following considerations, which are closely tied to *t*-Student-tests. Since the value *α* was set as 0.05 for these calculations, the zero hypothesis is rejected if Pearsons coefficient approach is ±1 and if *p* is lower than*   α*, thus leading to the conclusion that there is indeed relevant statistical correlation between the two variables under analysis. Otherwise, nothing be stated anything regarding the existence of the statistical correlation. From this point of view, major conclusions from Figures 6–10 and Table 3 may be summarized as follows.

- There is a significant statistical correlation between ST (AANSA) and MIB, at the channel level (see second line of Table 3).
- There is a very strong and significant statistical correlation between ST (AANSA) and MFR, MBR and MIB, at the experiment level (see third line of Table 3).
- There is a very strong and significant statistical correlation between ST estimated for the AANSA signals and the ST estimated for ISI time series, even if their absolute amplitudes are different from each other.

Particularly regarding the first conclusion above, based on Table 3 and on a linear regression model that can be derived from Figure 7, it is possible to establish the following analytic expression:

(8) $$\text{ST}\;\left(\text{AANSA}\right)≅0.6^{*}\text{MIB}.$$

In fact, the value 0.6 was obtained from Figure 7, which considers the context of each experiment, all channels.

There are, however, several relevant issues involving the results of the statistical analysis presented in Figures 6–10 and Table 3. Firstly, they are related to quantities which present different physical meanings and measurement unities. Secondly, the ST amplitude variation is very little when compared to the amplitude variation of the other quantities in Table 3. Finally, windowing performed for classical spike analysis does not match exactly that one used for DFA method.

Consider now results of Figure 5. As a general conclusion of Table 2, it can be stated that strong neurodynamical changes take place every 2.40 seconds in the cultured neurons. This conclusion agrees with previous results reported in [19], wherein the authors studied the statistical behaviour of the neuronal spontaneous activity of fusimotor neurons, measured in cats by in vivo experiments. In this paper, the stationarity profile of the signals is characterized as a second-order white noise, leading to the conclusion that spontaneous neural activity plays an important role on the stochastic resonance mechanism, which may be used to explain the underlying physiological process of this group of neurons.

Furthermore, the results shown in Table 2 also agree with the conclusions of [22]. In this article, the author analyzes the electrical activity of postsynaptic neurons from the somatosensorial cortex of rats, supposing that these cells are electrically stimulated by a specific protocol, according to Ornstein-Uhlenbeck Model, for which the presynaptic stimulation signal resembles to white noise. In the following, the statistical characterization of the postsynaptic response is accomplished based on the classical interspike interval time series. For this last signal, the stationarity time was estimated as about 2 seconds. It should be pointed out that such behavior takes place during all times of the experiment, until the end of the stimulation protocol carried out by the author. 

According to Table 2, the average-ST values for each experiment do not present significant difference at the time scale of 2 seconds, disclosing a similar stationarity pattern in the four experiments. The major differences between these average-ST amplitudes lie within the millisecond scale. Furthermore, as reported in [21], the four experiments were performed in a consecutive way, using the same cultured neurons, so that the second one was carried out after the first one, and so on. Based on this context, Table 2 points out that the average-ST amplitudes increased slightly as the experiments were performed, so that the average stationarity profile of the signals kept almost constant in time.

Figure 5 shows that there is a spatial distribution of the overall-average ST, so that it is possible to establish relations between specific groups of electrodes and the overall-average ST amplitudes. For example, regions K1, K2, and K3 in Figure 5 may be considered quite confined spaces, each of them connected with specific groups of electrodes, so that there is no overlapping involving such regions. In consequence, K1 characterizes regions with strong statistical variation, since its overall-average ST amplitude is minimum; whereas, K2 groups electrodes for which signals present a more stationary behaviour. On the other hand, region K3 is characterized by an intermediate statistical-variation profile. Consequently, the neural spontaneous activity of a cellular culture may present different statistical variation profiles, pointing out that electrical activity throughout the electrodes is structurally different. In the context of Figure 5, intermediate time variations may be considered the major average stationarity profile associated with the ensemble of signals recorded by MEA electrodes.

Figure 5 points out that difference between time-variation characteristics along the MEA lies within the millisecond scale, which suggests that such time variations are due to physiological processes connected to fine-tuning cellular mechanisms [22]. In brief, the overall-average ST of MEA electrodes could result from physiological phenomena. To our knowledge, the biological concepts that could be used to explain the ST are based on the dynamics action of the adaptation/facilitation components in different time scales, providing the modulation of neural responses after an electrical stimulus [22, 23]. 

Among these components, there are those characterized by a quick dynamics, which can change in the scale of hundreds of milliseconds, known as “brief components”. There are also other ones, presenting slower action, which will influence neurophysiological phenomena in the time scale of seconds [22], called “later adaptation”. According to results of Table 2, the average stationary behaviour of MEA signals is controled by the later adaptation, since all the overall-average STs lie within the range 2 seconds; whereas, the brief component can be considered responsible for the differences between the stationarity profiles in each electrode, in terms of the time scale of milliseconds, as shown in Figure 5(a). Even if the physiological mechanisms underlying the later adaptation have not been completely established yet, studies explain this phenomenon by the slow inactivation of the sodium channels, including also the influence of the brief facilitation component [23]. Although results depicted at Table 2 and in Figure 5 suggest strong evidence supporting the role of the physiological mechanisms previously described, it is not possible to establish a definite explanation on ST physiology associated with the cellular culture measured by the MEA device. 

Finally, comparing the results and discussions in this paper, in [19] and in [22], it can be noticed that the overall-average ST amplitudes converge almost to the same magnitude of 2–5 seconds, for several types of neuronal cells: fusimotor neurons of cats [19], somatosensorial cells of rat cortex [22], and cortical neurons of rat embryos** (**18 days of life**)**; the last ones are analyzed in this paper. At the same time, the white noise theory, associated with the ionic-channel dynamics, also emerges in an outstanding way, since it was used for explaining the results in [19] by the stochastic resonance theory, for the experimental protocol carried out in [23] and for the statistical characterization accomplished in [24]. This last paper employs the concept of white noise in the context of high-order statistical theory.

## 5. Conclusion and Future Work

The upgraded DFA method allowed the detection of nonstationarities of the neural-culture signals throughout the time. Our proposition enables a more accurate and systematic detection of signal ruptures, if compared to the classical straight-line rough approach [19, 20]. 

Results based on experiments and on a rigorous statistical analysis discussed the relationships among the ST (which was estimated for both AANSA signals and ISI time series) and the classical spike analysis quantities MIB, MFR, and MBR, which are generally calculated to assess the global physiological state of the culture. It was shown that ST (AANSA) does not present any correlation with both MFR and MBR. However, ST (AANSA), ST (ISI), and MIB (which is also calculated from ISI) do present a strong statistical correlation, so that it is possible to estimate MIB(ISI) as the ST (AANSA) divided by 0.6, as shown in (8). Notice that since the AANSA signal is composed of basal activity, bursts, and spikes, then indeed bursts may be considered a kind of “non-stationarity”, since they involve significant signal amplitude and frequency changes. In consequence, all these results suggest that it is possible to perform neural coding analysis based just on the absolute amplitude of the neural spontaneous activity, instead of considering the interspike interval (ISI) time series, currently used by classical systems. It should be noticed that ISI signals do not consider data present in the basal activity existent between spikes, thus losing (maybe) important biological information. In addition, ST estimation may also avoid inaccuracies and the high computational complexity associated to spike detection, which fulfils the requirements imposed by neuroprosthesis clinical use in terms of biocompatibility.

The upgrade on the DFA technique proposed in this paper leads to the estimation of the overall-average ST within the range 2-3 seconds, pointing out that the optimal windowing of signals arising from the neural culture is about 2-3 seconds. According to literature [19, 22], such result provides an accurate windowing, which is required by the classical methods of spike detection and classification for an efficient operation. Furthermore, it was shown that cellular culture signals can present different characteristics in its electrodes, which of course may be associated to different physiological behaviours. Particularly, for the signals considered in this paper, the average level of time variations may be considered intermediate, which defines the average non-stationary profile. Numerical results and previous work in literature suggest that the mechanism of later adaptation defines the global behavior of MEA signals; whereas, the brief facilitation component is responsible for the slight differences associated with particular non-stationary features of each electrode.

Future work involves the use of the upgraded DFA method for the development of efficient spike classification and neural connectivity techniques, based on the concept of ST. White-noise theory plays also an important role according to previous works of literature, suggesting its association with DFA. Finally, deeper studies about the role of ionic channels involved in the physiological mechanisms of adaptation/facilitation should be performed to evaluate the association of these channel dynamics with the ST profile of the neuronal culture. 

## Figures and Tables

**Table 1:** Average results of ISI analysis.

Number of spikes/second within one burst	Average duration of one burst	Average number of spikes during one burst	Average duration of one spike within the burst
78.02	161.16 seconds	12570	12.82 milliseconds

**Table 2:** Average values for each experiment, considering all the 60 channels, based on (7) and classical spiking processing.

Experiment number	ST [s] – AANSA signals	ST [s] – ISI time series	MIB [s]	MFR [spikes/s]	MBR [bursts/min]
1	2.29 ± 0.06	3.10 ± 0.04	3.26 ± 0.01	10.01 ± 0.06	19.07 ± 0.06
2	2.41 ± 0.06	4.93 ± 0.02	5.03 ± 0.02	6.63 ± 0.01	13.63 ± 0.09
3	2.50 ± 0.06	6.12 ± 0.01	6.44 ± 0.02	5.69 ± 0.03	11.77 ± 0.02
4	2.42 ± 0.06	5.53 ± 0.03	5.61 ± 0.01	7.17 ± 0.02	13.57 ± 0.03
Overall average	2.40 ± 0.06	4.92	5.09	7.38	14.51

*Remarks on abbreviations*: AANSA = Absolute-Amplitude Neural Spontaneous Activity; ISI = Interspike Interval; MIB = Mean InterBurst Interval; MFR = Mean Firing Rate; MBR = Mean Burst Rate.

**Table 3:** Pearson Correlation Coefficients (*r*) among ST and other classical measures, relationship with Figures 6–10. *α* = 0.05.

Comparison in terms of	*r* involving ST and MFR [spikes/s]	*r* involving ST and MBR [burst/min]	*r* involving ST and MIB [s]	*r* involving ST (estimated for AANSA signals) and ST (estimated for ISI time series)
Channels, considering four experiments	*r* = −0.0111;	*r* = 0.1591;	*r* = 0.5956;	*r* = .9654
	*P* = .9334 Figure 6	*P* = 0.2285	*P* < .0001 Figure 7	*P* = .0118
Experiments (5 minutes, all channels at a time)	*r* = −0.9721;	*r* = −0.9784;	*r* = 0.9891;	*r* = 0.9792;
	*P* = .0279 Figure 8	*P* = .0216	*P* = .0109 Figure 9	*P* = .0208 Figure 10
